# Carcinosarcoma of the Rectum: Report of a Rare Colorectal Malignancy and Review of the Literature

**DOI:** 10.1155/2015/412918

**Published:** 2015-11-17

**Authors:** Alexis Sudlow, Ming Ho Liu, Geoffrey Waters, Vamsi R. Velchuru

**Affiliations:** ^1^Department of Colorectal Surgery, James Paget University Hospital, Gorleston NR31 6LA, UK; ^2^Department of Histopathology, Norfolk and Norwich University Hospital, Norwich NR4 7UY, UK

## Abstract

Carcinosarcoma (CS) is a rare mixed mesodermal malignancy most commonly affecting the female reproductive organs, respiratory tract, head, and neck. Though infrequent, it may affect the gastrointestinal tract, most often the oesophagus and only very rarely the rectum. Histologically, it is composed of two distinct elements of epithelial and mesenchymal origin. Clinically, it is a very aggressive tumour with many patients presenting with metastatic lymph nodes or distant metastases at the time of diagnosis. Prognosis is poor despite intervention with the majority of patients dying within six months. Due to the rarity of this condition, there are no specific treatment guidelines presently available. We describe the case of an 80-year-old patient with carcinosarcoma of the rectum with discussion of the immunohistochemistry and review the available literature pertaining to this rare presentation.

## 1. Case Report

**Figure 1:**
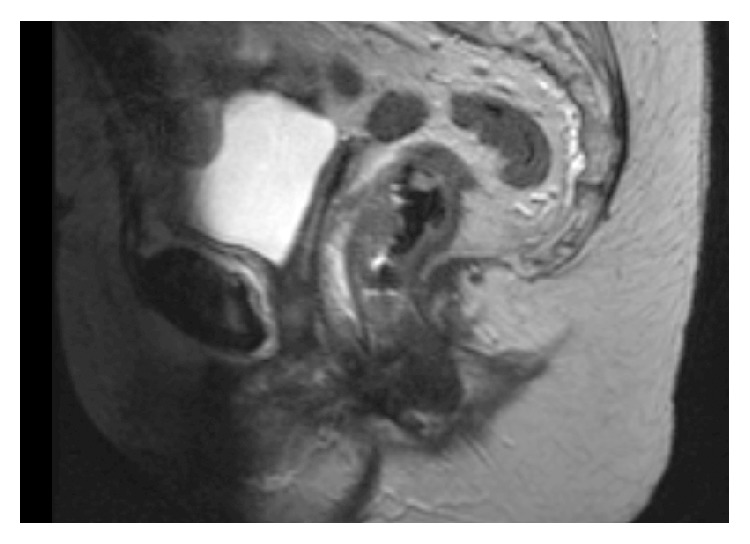
Staging MRI demonstrating low rectal mass, invading potential anterior resection margin.

An 80-year-old female presented with a four-week history of fresh rectal bleeding, associated with poor appetite and weight loss. There were no other symptoms relating to change in bowel habit. Past medical history included ischemic heart disease, atrial fibrillation, hypertension, Parkinson's disease, and hysterectomy several years previously for benign disease. Abdominal examination was unremarkable; however, a low rectal tumour was palpated anteriorly on digital rectal examination. Subsequent colonoscopy showed a large solitary polypoidal growth in the lower rectum. Biopsy of the lesion provided a histological diagnosis of CS based on the presence of two clearly identifiable components on HPE. The tumour consisted of malignant glands set within an atypical stroma consisting of fascicles of spindle cells with thin, oval tapered nuclei and eosinophilic cytoplasm with perinuclear vacuolation. The stromal spindle cells showed florid mitotic activity. Further immunostaining confirmed the nature of the two components with the glandular tissue staining positively for CK20 and CEA. The stromal component did not stain positively for epithelial markers and was negative for cytokeratins. Staging computed tomography (CT) scan of the chest, abdomen, and pelvis and pelvic magnetic resonance imaging (MRI) were performed (Figure 1). A low rectal tumour at 4.2 cm from the dentate line was seen with extramural invasion to the potential anterior resection margin. Three suspicious lymph nodes were noted and the radiological staging was T3 N1 M0.

**Figure 2:**
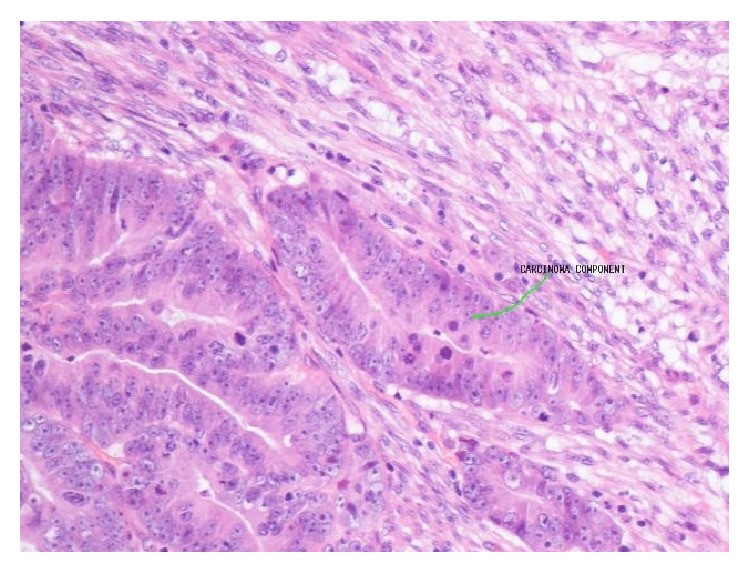
HPE showing distinct areas of carcinoma visible in resected specimen. The carcinomatous segment is well differentiated.

**Figure 3:**
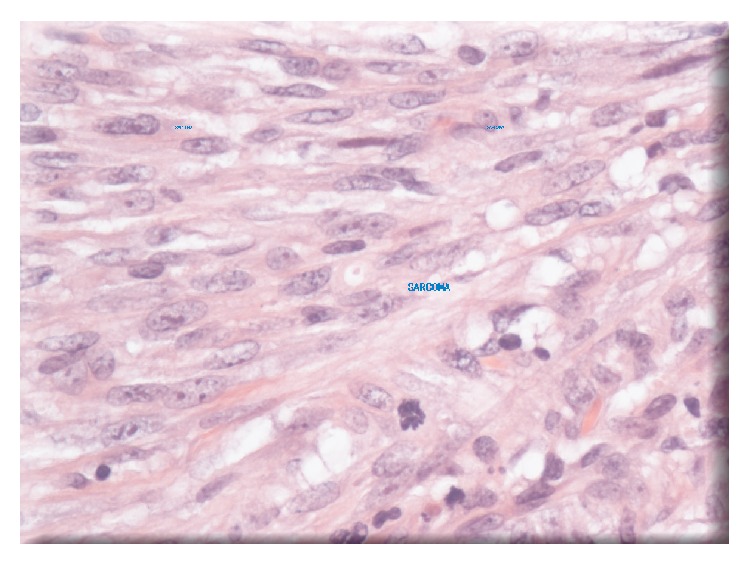
Sarcomatous component visible on HPE.

Following a five-week course of preoperative radiotherapy, the patient underwent radical laparoscopic-assisted abdominoperineal excision of the rectum (APER) with curative intent. Postoperative recovery was uneventful. Histological examination of the resected tumour revealed small amount of residual disease consisting largely of a poorly differentiated adenocarcinoma with a cellular stroma. The malignant tumour showed distinct carcinomatous (Figure 2) and sarcomatous (with leiomyosarcomatous differentiation) (Figure 3) components. There was extramural vascular invasion, as well as lymphatic and intramural venous invasion. Large areas of the tumour were necrotic with residual granulation tissue and fibroblastic response replacing the original tumour. All eight lymph nodes showed reactive changes only with small areas of fibrosis present. Histopathological staging was ypT3 N0 Mx, Duke's B2 poorly differentiated residual carcinosarcoma.

**Figure 4:**
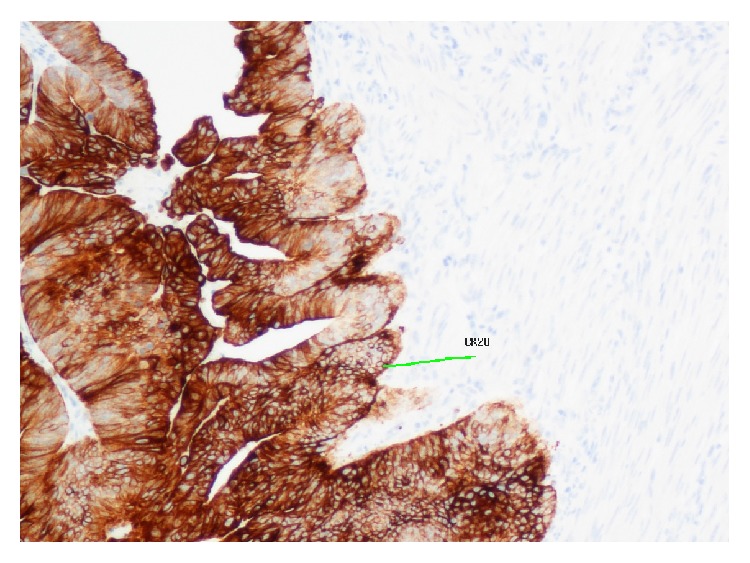
Immunohistochemistry showing positive staining for CK20 in the carcinomatous component.

**Figure 5:**
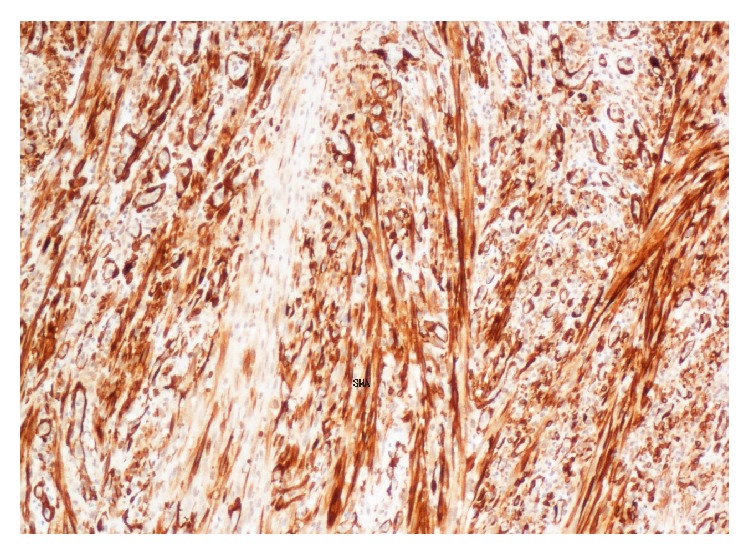
Positive staining for SMA in the sarcomatous component.

**Figure 6:**
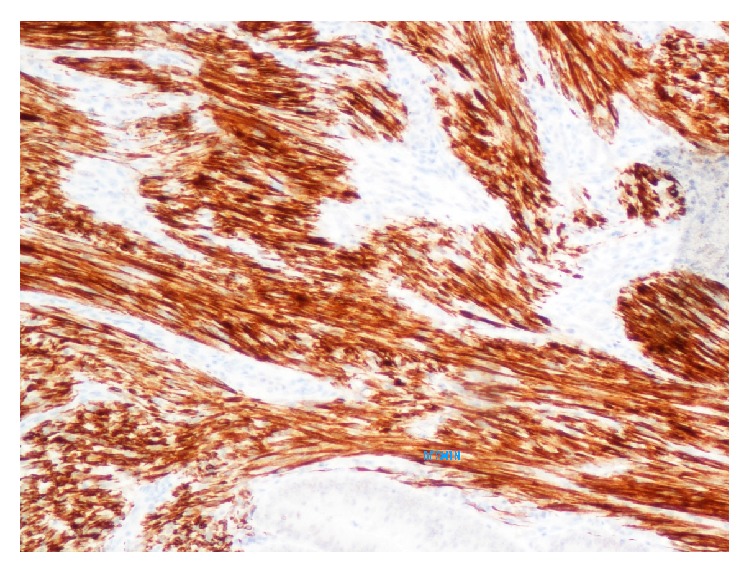
Positive staining for desmin in the sarcomatous component.

**Figure 7:**
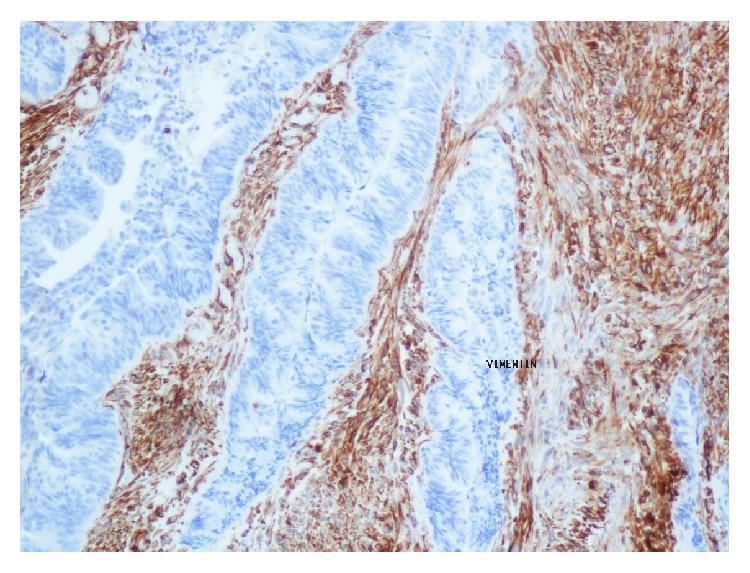
Positive staining for vimentin in the sarcomatous component.

Given the heterogeneous nature of the tumour, the two components showed distinct immunohistochemistry. The epithelial component stained positively for CK20 (Figure 4) and CEA while the sarcomatous component was positive for SMA (Figure 5), desmin (Figure 6), and vimentin (Figure 7).

Eight-month follow-up did not show any evidence of recurrence and she was clinically well; however, she subsequently developed locoregional recurrence with pulmonary metastases at 16 months. In view of her frailty and significant cardiac risk factors, she was not considered for chemotherapy and succumbed to the disease 25 months after surgery.

## 2. Discussion

The term carcinosarcoma is used to describe a rare mixed mesodermal malignancy consisting of an epithelial component most often a mid to high grade adenocarcinoma and a sarcomatous element of mesenchymal origin which may be differentiated or undifferentiated [1]. CS displays a preponderance for the female reproductive tract, most often the ovaries and uterus, but is also found within the head, neck, and respiratory and gastrointestinal tract [2, 3]. The first case report describing the appearance of CS within the bowel was published in 1986 by Weidner and Zekan, and since then, there have been a further 19 cases with only four specifically involving the rectum [4] (Table 1). Review of previously reported cases of CS within the rectum suggests it affects a slightly older population of patients with a mean age of 75.2 years as compared to 67.6 years in a review including all cases of CS involving the colon.

The presentation and malignant behavior of CS is very similar to that of adenocarcinoma with the carcinomatous component showing a strong tendency to metastasise to lymph nodes and distant sites whereas the sarcoma plays only a very minor role in metastatic spread [5]. Patients may report symptoms such as rectal bleeding [1, 6, 7], weight loss [8], and abdominal pain [9] and obstructive symptoms [10].

The histogenesis of carcinosarcoma is poorly understood and multiple hypotheses have been proposed. Further characterization of the nature of the tumour itself has been achieved through immunohistochemistry, confirming the presence of distinct components. The most commonly observed pattern of staining is reactivity in the adenocarcinomatous component to the epithelial markers, CK20 and CEA [5, 9]. The sarcomatous cells frequently stain positively for vimentin, desmin, and SMA [1, 4, 5].

Within the large bowel, there are reports of carcinosarcoma from the caecum to the rectum; however, there does not appear to be any significant difference in terms of the potential to metastasize or the overall prognosis [9]. A review of 18 previous cases found metastatic disease in seven patients with the most common sites being liver, lymph nodes, and omentum [5] and, in the present case, lung. Due to the rarity of CS within the large bowel, no specific treatment guidelines exist; however, evidence from available case reports would suggest that it should be treated in a similar fashion to colonic adenocarcinoma with surgical resection and removal of suspicious lymph nodes being the mainstay of treatment.

Prognosis of CS within the colon is poor with the longest reported survival being 49 months [4]. Rectal CS carries even worse prognosis with all cases prior to the current case dying between five weeks and six months [6, 10–12] (Table 1). Given the rarity of the condition, specific prognostic factors have not been clearly identified; however, suggested indicators include size, stage, lymphatic or vascular invasion, and histology of the carcinomatous component [9]. The histology of the sarcomatous component does not appear to affect prognosis in cases involving other organs; however, it is unclear whether this holds true within the colon [12–14].

Evidence supporting the addition of adjuvant chemoradiotherapy in terms of improving long term outcome is lacking; however, the addition of RT for the patient in the current case did result in downstaging of the disease, allowing for resection with curative intent. Chemotherapy was not a viable option due to her significant cardiac comorbidities. As was done in this patient, strict follow-up is essential given the aggressive nature of this malignancy to detect any local recurrence or metastasis. Although this patient succumbed to the disease after 25 months, she remained disease-free until 16 months post-op which is significantly longer than all other reported cases of rectal carcinosarcoma.

## 3. Conclusion

Rectal carcinosarcoma is a rare, aggressive mixed type of malignancy with mesenchymal and epithelial components. Immunohistochemistry is the gold standard for diagnosis, identifying two key features: differential staining of the distinct components and the lack of staining for epithelial markers in the sarcomatous component. Aggressive treatment similar to adenocarcinoma protocols with strict follow-up can improve survival rates.

## Figures and Tables

**Table 1:** Patient characteristics of previous case reports of rectal carcinosarcoma.

Study	Age/sex	Metastasis	LN involvement	Adjuvant Rx	Survival
Roncaroli	71/F	N	Y	Pre-op RT	6 months
Takeyoshi	82/M	Skin			6 months
Tsekouras	60/M	Local recurrence, pulmonary + hepatic metastasis	Y	Post-op chemo/RT	6 months
Kolodziejczak	83/M	None	N	None	5 weeks
Present case	80/F	Local recurrence, pulmonary	N	Pre-op RT	25 months
